# Designing Microblog Direct Messages to Engage Social Media Users With Suicide Ideation: Interview and Survey Study on Weibo

**DOI:** 10.2196/jmir.8729

**Published:** 2017-12-12

**Authors:** Ziying Tan, Xingyun Liu, Xiaoqian Liu, Qijin Cheng, Tingshao Zhu

**Affiliations:** ^1^ Institute of Psychology Chinese Academy of Sciences Beijing China; ^2^ Harvard Graduate School of Education Harvard University Cambridge, MA United States; ^3^ Department of Psychology University of Chinese Academy of Sciences Beijing China; ^4^ Hong Kong Jockey Club Center for Suicide Research and Prevention The University of Hong Kong Hong Kong China (Hong Kong)

**Keywords:** microblog direct message, social media, suicide prevention

## Abstract

**Background:**

While Web-based interventions can be efficacious, engaging a target population’s attention remains challenging. We argue that strategies to draw such a population’s attention should be tailored to meet its needs. Increasing user engagement in online suicide intervention development requires feedback from this group to prevent people who have suicide ideation from seeking treatment.

**Objective:**

The goal of this study was to solicit feedback on the acceptability of the content of messaging from social media users with suicide ideation. To overcome the common concern of lack of engagement in online interventions and to ensure effective learning from the message, this research employs a customized design of both content and length of the message.

**Methods:**

In study 1, 17 participants suffering from suicide ideation were recruited. The first (n=8) group conversed with a professional suicide intervention doctor about its attitudes and suggestions for a direct message intervention. To ensure the reliability and consistency of the result, an identical interview was conducted for the second group (n=9). Based on the collected data, questionnaires about this intervention were formed. Study 2 recruited 4222 microblog users with suicide ideation via the Internet.

**Results:**

The results of the group interviews in study 1 yielded little difference regarding the interview results; this difference may relate to the 2 groups’ varied perceptions of direct message design. However, most participants reported that they would be most drawn to an intervention where they knew that the account was reliable. Out of 4222 microblog users, we received responses from 725 with completed questionnaires; 78.62% (570/725) participants were not opposed to online suicide intervention and they valued the link for extra suicide intervention information as long as the account appeared to be trustworthy. Their attitudes toward the intervention and the account were similar to those from study 1, and 3 important elements were found pertaining to the direct message: reliability of account name, brevity of the message, and details of the phone numbers of psychological intervention centers and psychological assessment.

**Conclusions:**

This paper proposed strategies for engaging target populations in online suicide interventions.

## Introduction

The number of people with suicide ideation has increased at an alarming rate, and this population suffers both physically and mentally. An estimated 1.53 million people will die from suicide, and even more people will contemplate suicide in the year 2020 on a global scale [1]. Moreover, Asia is the region that has the largest numbers of suicides, and China and India alone occupy 30% of all cases of suicide worldwide, not to mention underreported incidents of suicide. By definition, suicide is a self-initiated act to end one’s own life [2], and suicide ideation relates to thoughts that may lead to suicide or nonfatal outcomes. Suicide and suicide ideation are probably complex actions that are associated with the interaction of various factors; it is likely that patients with suicide ideation suffer greatly. According to Gvion and Apter [3], almost 90% of cases of patients with suicide have psychiatric disorders, and those with suicide ideation also experience unbearable mental pain. Not only does the patient suffer, his or her family members and friends are also faced with enormous mental, emotional, and physical stress. In order to alleviate this pain, suicide intervention is vital for this target population.

Substantial research has been conducted for the intervention. Suicide hotlines and intervention centers are the most prevalent ends for suicide prevention. However, De Leo et al [4] suggest that only 20% of patients are willing to seek help and receive treatment. The other 80% are not completely opposed to receiving help but have not proactively asked for help. In other words, this passive help-offering attitude tends to make suicide intervention less available for the population with suicide ideation, who in reality need no less prompt treatment than any other patients.

Machine learning’s suicide risk identification on microblogs provides the possibility for proactively finding individuals with suicide ideation. Kessler and Walters [5] find that young people, especially the 24-year-old group, have the highest rate of suicide. Many young people prefer to use the Internet to search for health information, including seeking treatment in early stages of suicide ideation [6]. In China, Sina Weibo is a microblogging platform that enables Chinese media users to disseminate and acquire information and is mainly used by young people. Many users have expressed suicide ideation and have broadcasted suicide attempts on this social media [7-11]. Included on the platform, Direct Message is another vital method of Chinese microblogging. It is similar to Twitter’s Direct Message, and users interact with others by sending private messages. Through proactive suicide risk identification, emergent help text via direct message could be provided to those with suicide ideation. Doing so may in turn increase the efficacy of suicide intervention.

The aim of this research was to engage the target group with direct messages by investigating the principles of designing both appropriate format and content of the information from the patients’ perspective. Since the target population is often opposed to active help-seeking, it is crucial to rely on the intended users at all levels of the design process. Specifically, we proposed to survey the population with current suicide ideation about acceptable subject lines and intervention descriptions for gaining a better understanding of how to proactively provide the target audience with accurate and effective information.

## Methods

### Overview

Two studies were conducted in this research. The first one examined user preferences and attitudes toward direct messages. Two group interviews were conducted and participant suggestions toward direct messages were solicited. To ensure the reliability and consistency of the results, another group of participants attended an identical group interview. A prototype of the direct messages was then integrated with the comments from the participants of the 2 interviews. In the second study, more patients were invited online to complete surveys about their preferences for direct messages as well as the prototype.

### Study 1

The purpose of this study was to examine user preferences and attitudes toward microblogging direct messages. Individuals were recruited via the Internet and asked to complete the Adult Suicide Ideation Questionnaire (ASIQ-4). The ASIQ-4 is a 4-item, self-reported questionnaire used for screening and measuring the severity of suicide ideation over the previous 12 months [12]. It is a short version of the Chinese ASIQ used in order to increase the efficacy of the screening. The ASIQ-4 uses a 7-point scale indicating the frequency of suicide ideation ranging from 0 (never had this thought) to 6 (almost every day). Participants with a score of more than 1 were eligible to attend a 2-hour group interview to provide feedback on key components of direct messages designed to provide in-time help.

In order to communicate with each participant effectively, 2 identical group interviews were conducted in the sequence of group 1 and group 2. Participants were invited on a first-come-first-serve basis, and each interview lasted 2 hours. A total of 8 participants completed the first interview, and 9 participants attended to the second interview anonymously (see Table 1).

**Table 1:** Demographic information of participants..

Variable		Group 1 (n=8)	Group 2 (n=9)
**Gender**			
	Male	5	4
	Female	3	4
Age (years), mean (SD)		23.13 (2.23)	23.22 (2.59)

As a last step, participants received a CNY ￥100 incentive after providing consent. All study procedures were approved by the Group Health Institutional Review Board.

Interviews were led by a doctor from Beijing HuiLongGuan Hospital, the most renowned center in China for suicide intervention. Two groups were presented with a direct message prototype which included general advice on suicide prevention and a link to psychological intervention hotlines. Since microblogging contains many messages to be read, it is important for the direct message to draw the attention of the user by understanding the target population’s attitude toward the message. The discussion includes the user preferences and attitudes toward direct message, as well as the advantages and disadvantages of the prototype.

### Study 2

The purpose of study 2 was similar to study 1: namely, to examine variables that contributed to drawing attention to online direct messages for suicide intervention. Unlike study 1, however, participants in study 2 were invited to complete an additional 10-question survey about the design of direct messages after the focused groups’ suggestions were integrated.

In total, 4222 Weibo users were invited to voluntarily take part in this study. None of these users had participated in study 1.

To find potential participants, we downloaded 65,352 comments (from March 2016 to September 2016) to the last tweet of a prominent blogger who had committed suicide, and 6 psychology graduate students were recruited to rank the risk level of these comments. The ranking standard consisted of a 4-point rating scale completed by each graduate student. The comment could be ranked as (0) No indication of suicide ideation and practical plan that contains the expression of death wishes or depression, (1) suicide ideation is detected while no detailed plan is made, (2) suicide ideation is detected while emergency intervention is not required (the content includes discussion of the action of death, death kit, death place and time, death will, etc), or (3) high risk of completing suicide, and emergency intervention is needed (detailed suicide plan or possibility of executing the plan within 1 or 2 weeks). After training, the consistency of the 6 psychology graduate student ratings reached a Cronbach coefficient of 0.85. For any user with risk level 3, a direct message was immediately sent out to offer emergency help.

Of 65,352 ratings collected, 8833 were labeled with suicide ideation, including level 1, 2, and 3. After amalgamating the overlapped microblog identifications, 4222 users were invited to complete the ASIQ-4 and the questionnaire about their suicide ideation anonymously through Psychological Map, the public account of the research group.

## Results

### Study 1

#### User Attitudes Toward Direct Messages

While 63% (5/8) were agreeable to reading all microblog private messages in the first interview, all 9 participants from the second interview (100%) intended to read the messages. All participants in both interviews claimed that they would be more vigilant if the sender were not an acquaintance, and they would like to pay more attention to the category of the account (whether the account is a marketing account) as well as the real purpose of the message. Two (2/8, 25%) appeared to read the private message only if the sender was an acquaintance, and 1 refused to read any messages in the first interview (see Table 2).

#### Online Interaction Through Direct Message

In the first interview, 38% (3/8) took the initiative to interact online using private chat, and in the second interview, 78% (7/9) used private chat for circumstances like greeting strangers.

#### Direct Message Checking Frequency

In the first interview, 75% (6/8) of participants claimed that they checked the private chat as long as they saw microblog notifications, and 13% (1/8) preferred to check messages on a daily basis. The rest (1/8, 13%) chose not to view the chats until the messages automatically popped up.

In the second interview, 44% (4/9) preferred to pay attention to the messages if they saw any notifications in their microblogs, 33% (3/9) chose to view messages on a daily basis, and 11% (1/9) checked messages on a weekly basis. Similarly, 11% (1/9) of respondents preferred not to see the messages unless they appeared on the front page (see Table 3).

#### Direct Message Length

In the first interview, 25% (2/8) believed that they would read the whole chat even if it was of great length. Another 13% (1/8) claimed to read lengthy chats only in the evening when he was the freest. The rest (5/8, 63%), read the messages based on their interest, demand, and the credibility of the sender despite of the length of the chat.

In the second interview, 11% (1/9) had no patience for reading long chats, and the rest (8/9, 89%) had the same attitude of reading the messages based on their interest, demand, and the credibility of the sender (see Table 4).

**Table 2:** User attitudes toward the microblog direct messages.

Variable	Group 1 (n=8)	Group 2 n=9)
Read all microblog private chats	5	9
Vigilant if the sender is not an acquaintance	8	9
Read the message only if the sender is an acquaintance	2	0
Refuse to read any messages	1	0

**Table 3:** Direct message checking frequency.

Frequency	Group 1 (n=8)	Group 2 (n=9)
As long as they see microblog notifications	6	4
Daily basis	1	3
Not to view unless the messages pop up	1	1
Weekly basis	0	1

**Table 4:** Direct message length preferences.

Variable	Group 1 (n=8)	Group 2 (n=9)
Read the whole chat regardless of the length	2	0
Read lengthy chats only in the evening	1	0
Read based on interest, demand, etc	5	8
No patience for long chats	0	1

#### Opinions Toward the Direct Message Prototype

Both groups felt the public account name, Psychological Map, was not clear, failed to embody public welfare, and was difficult for them to determine the nature of the account. To solve this problem, naming the account in a warmer way to help the target group feel the social care and support was advised. Another noticeable feedback was the need to highlight the aim of the text to make sure the target population knows that the account is for professional use and intends to support this group under privacy protection. In terms of the text length, shorter length tended to be more suitable for the audience. Both groups also were agreeable to the addition of psychological crisis intervention center phone numbers in the private chat content. A more customized design rather than general advice was expected from the second interview group.

### Study 2

#### Overview

**Figure 1:**
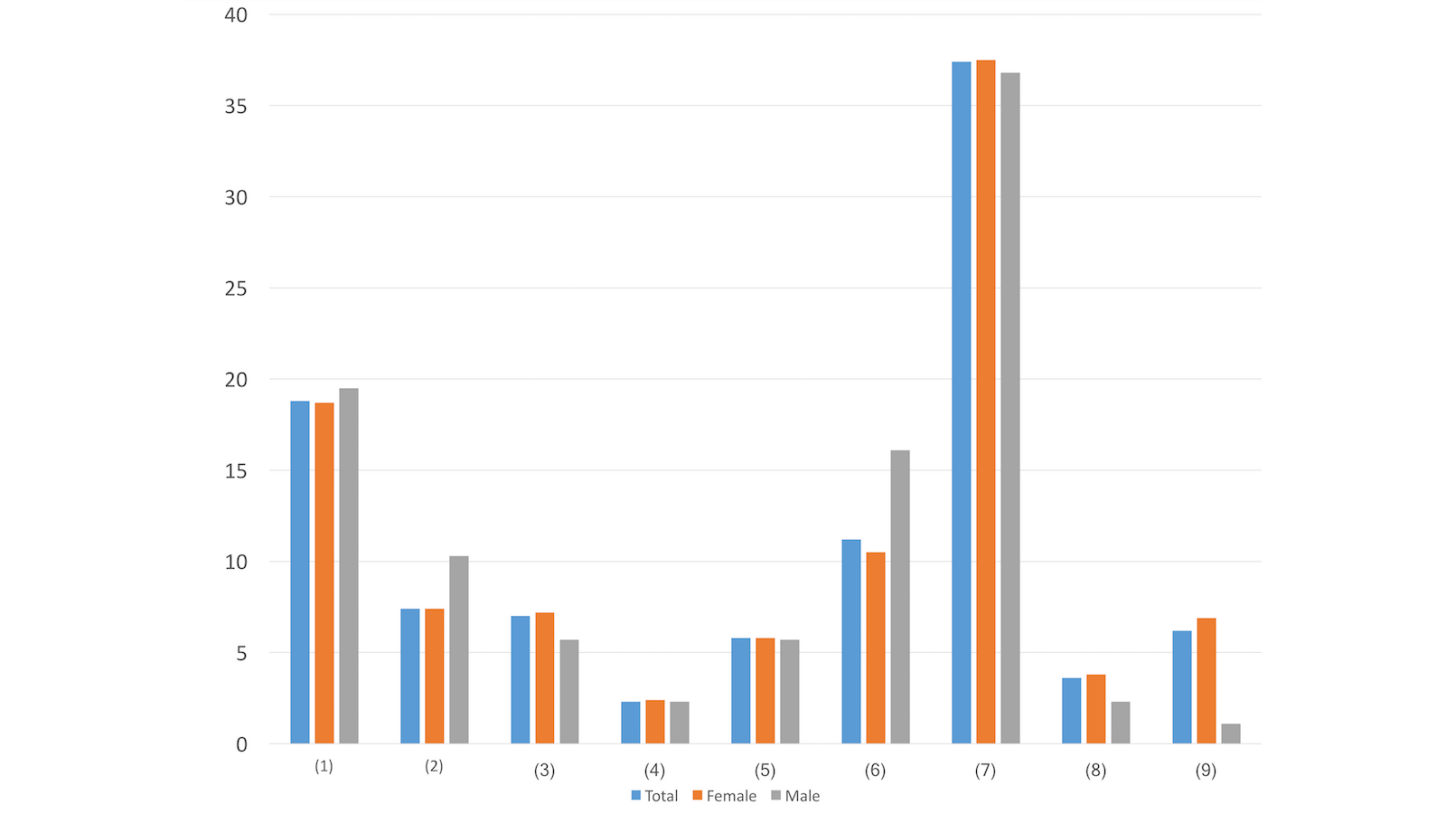
Results of study 2 question 1.

**Figure 2:**
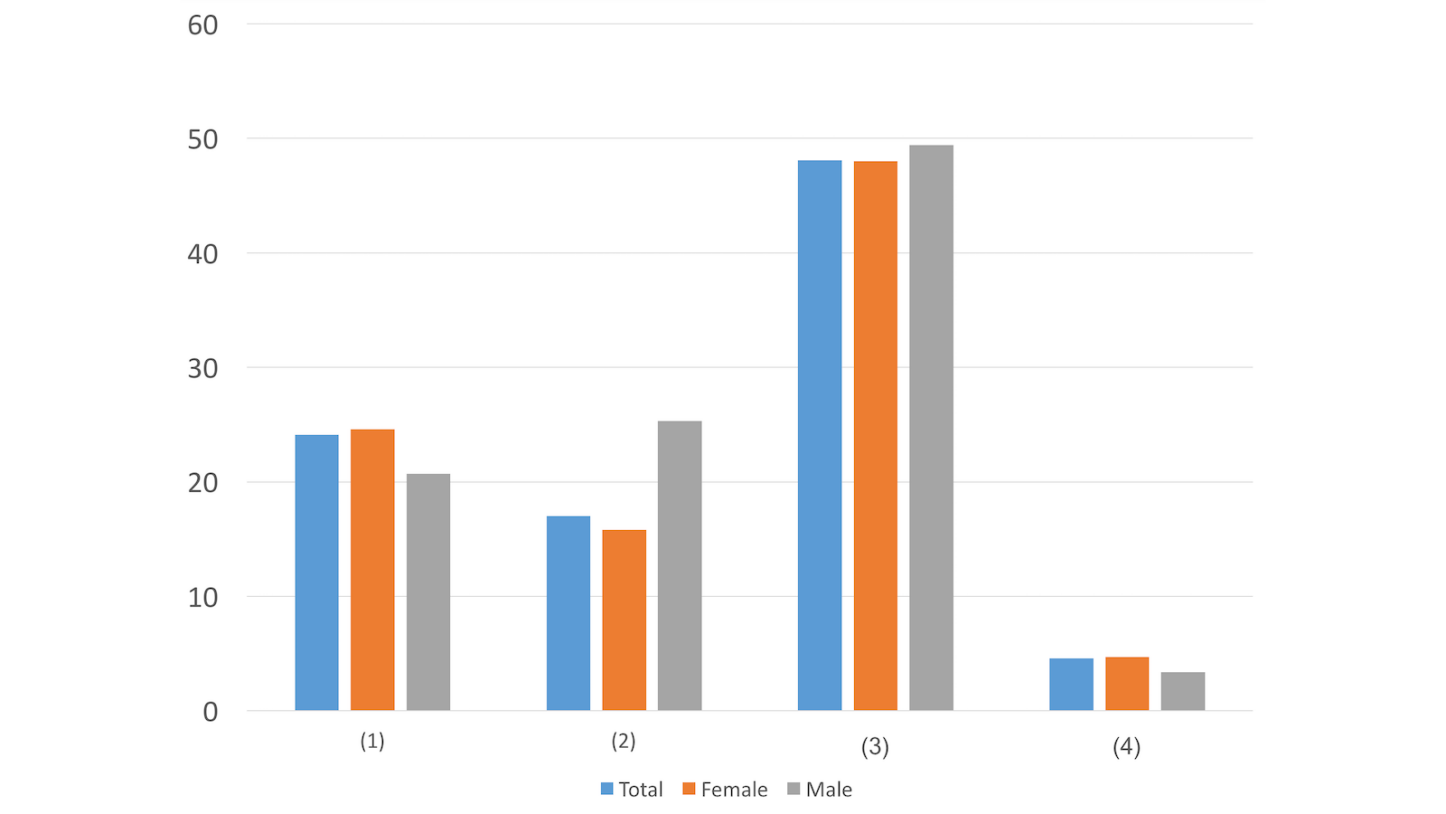
Results of study 2 question 2.

**Figure 3:**
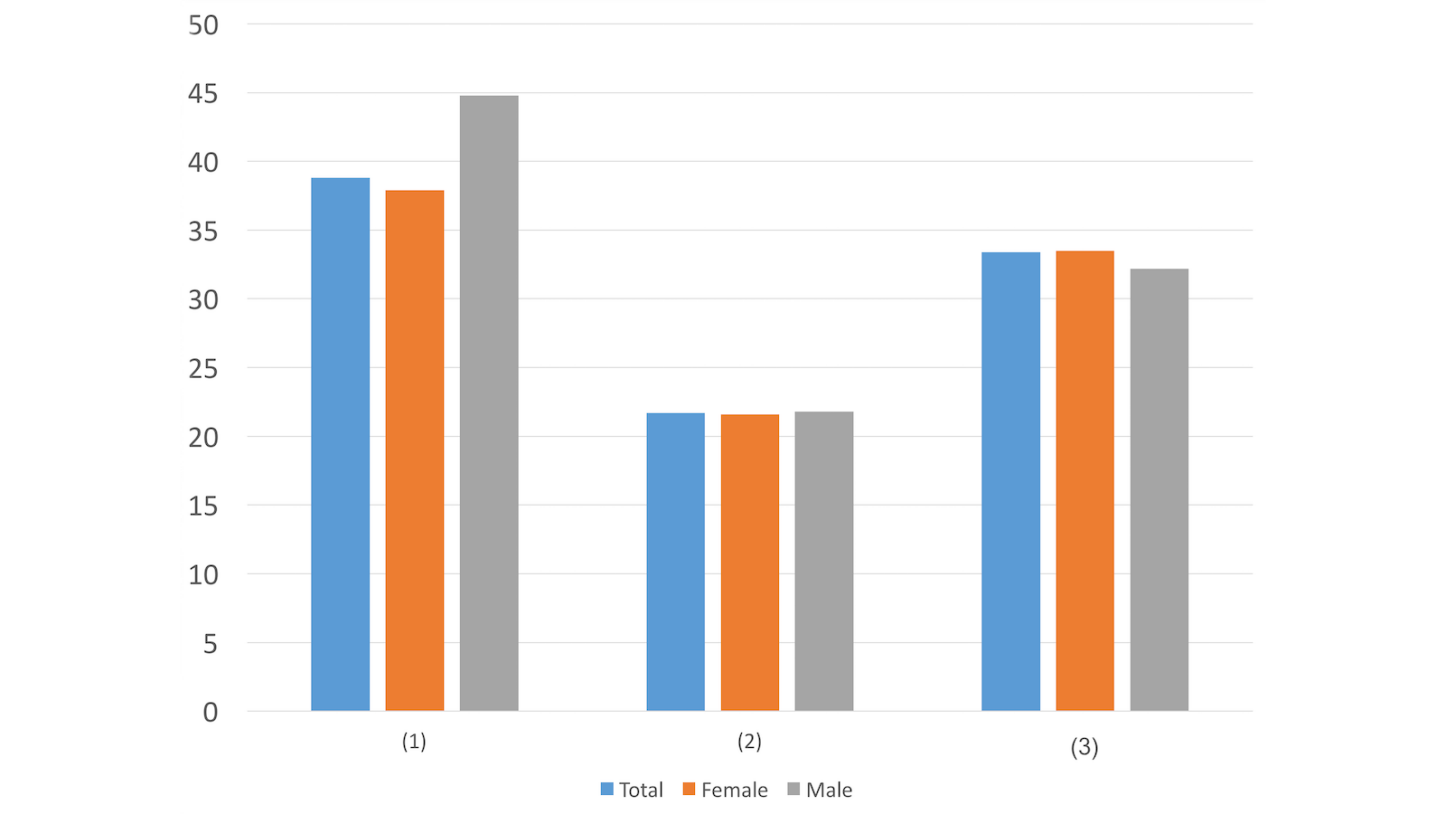
Results of study 2 question 3.

**Figure 4:**
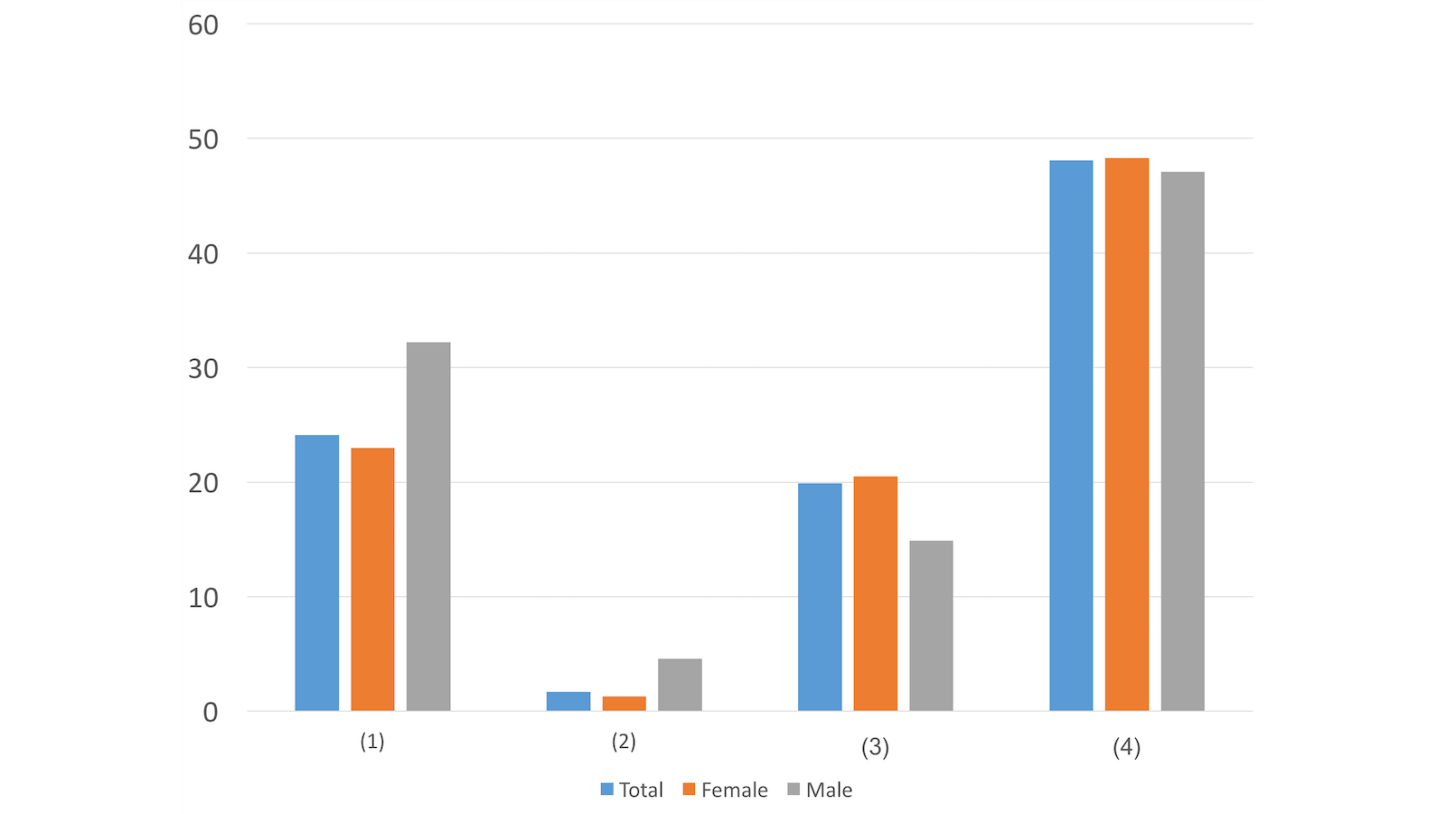
Results of study 2 question 4.

**Figure 5:**
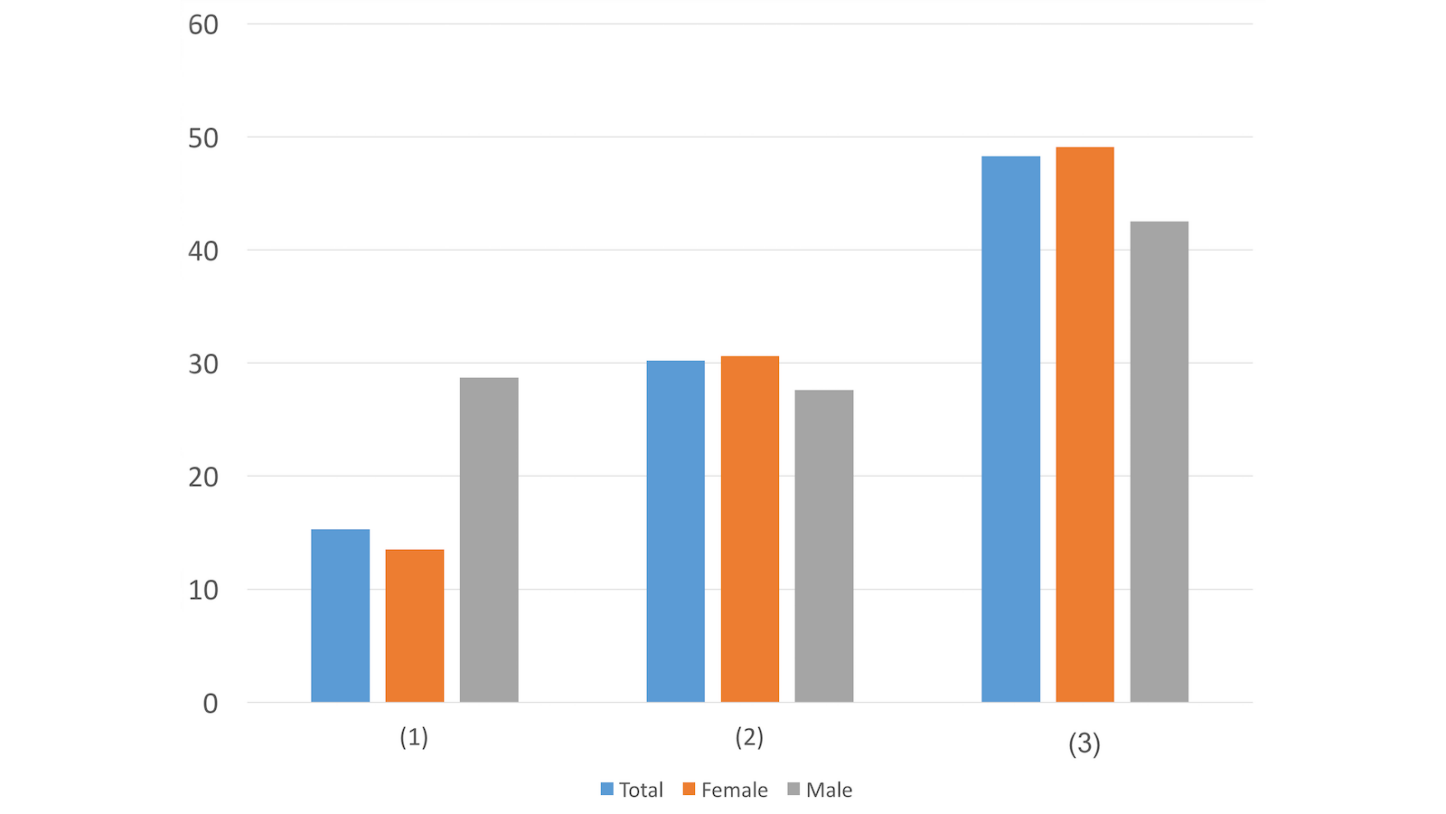
Results of study 2 question 5.

**Figure 6:**
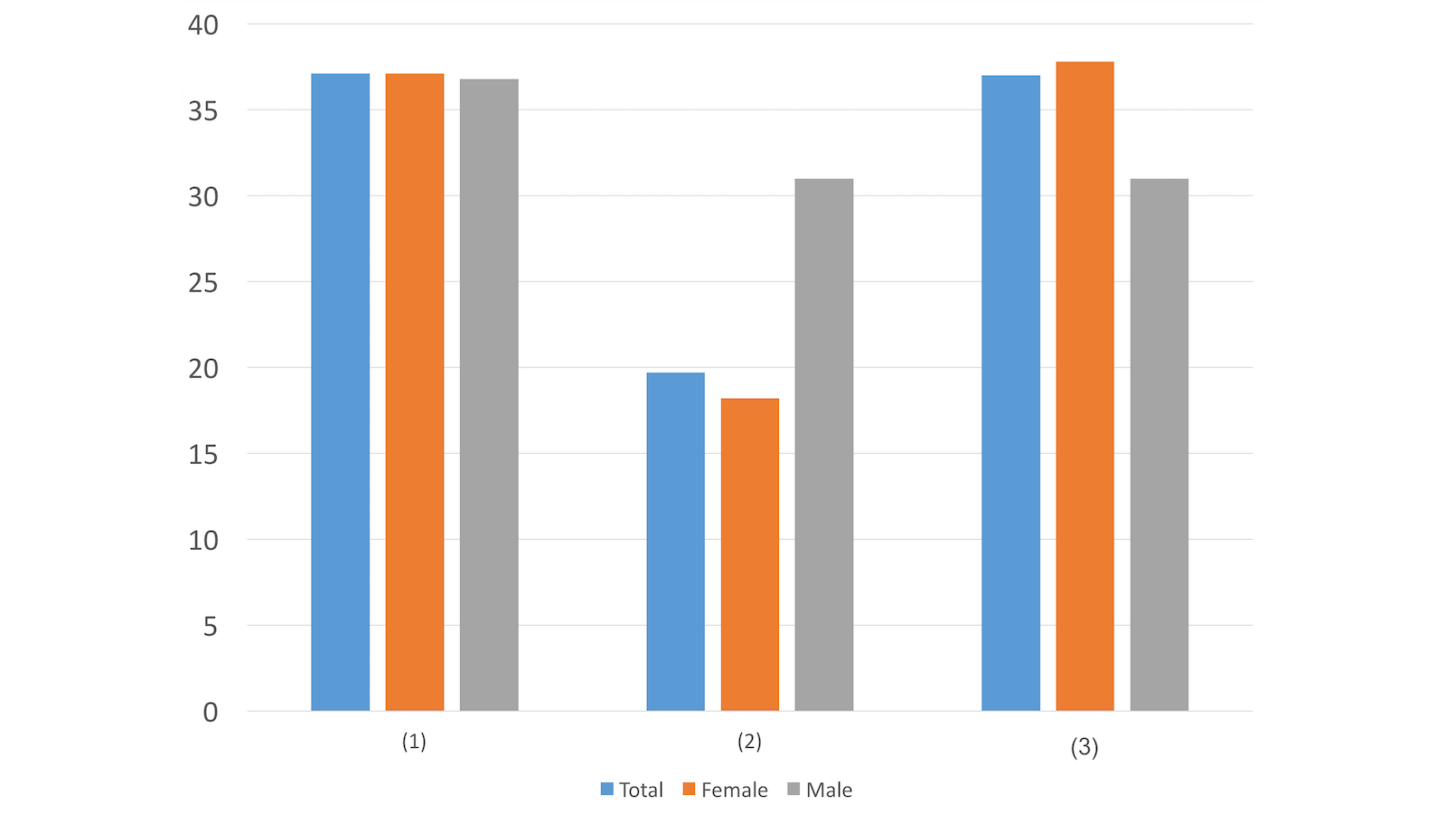
Results of study 2 question 6.

**Figure 7:**
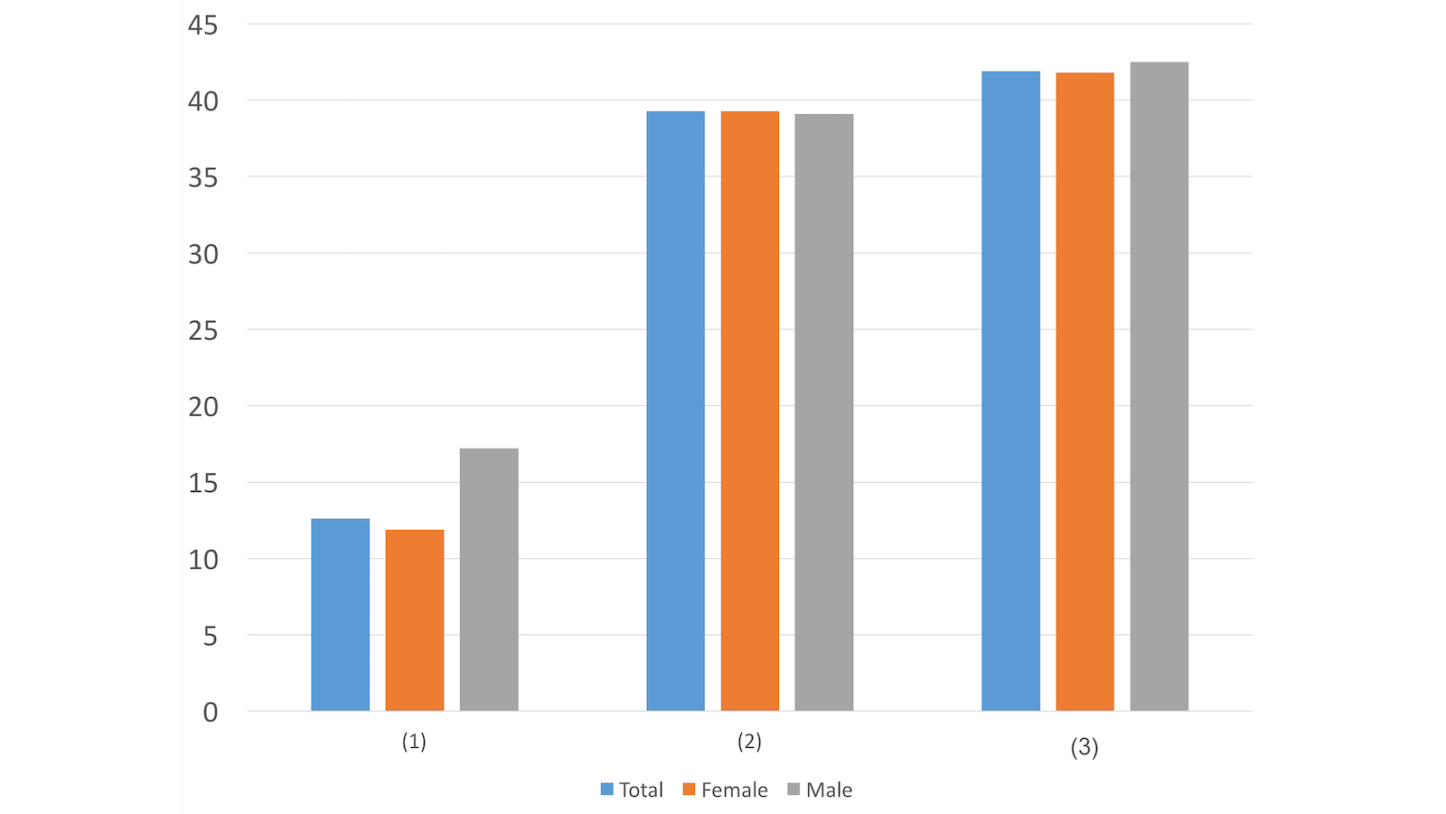
Results of study 2 question 7.

**Figure 8:**
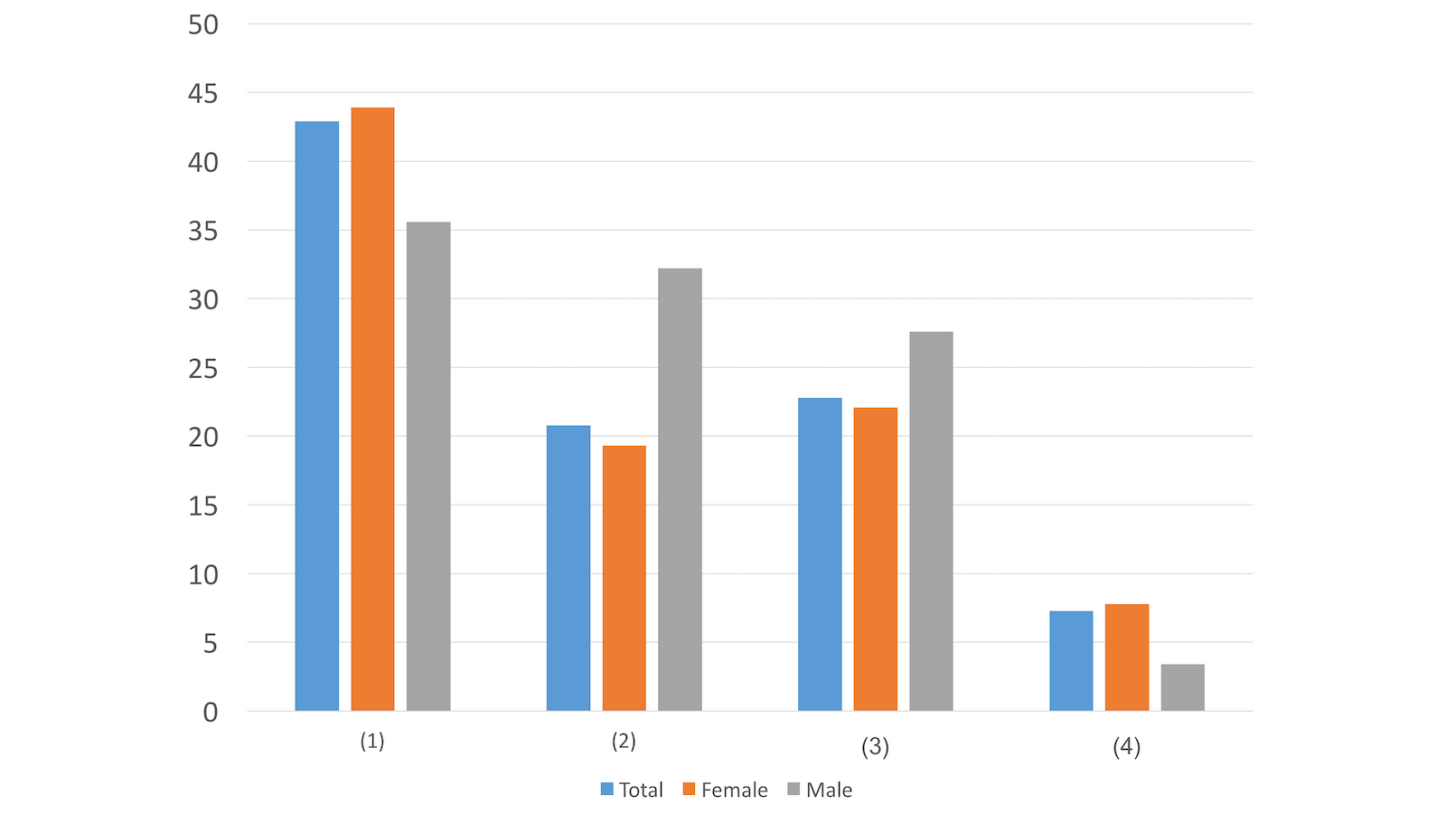
Results of study 2 question 8.

**Figure 9:**
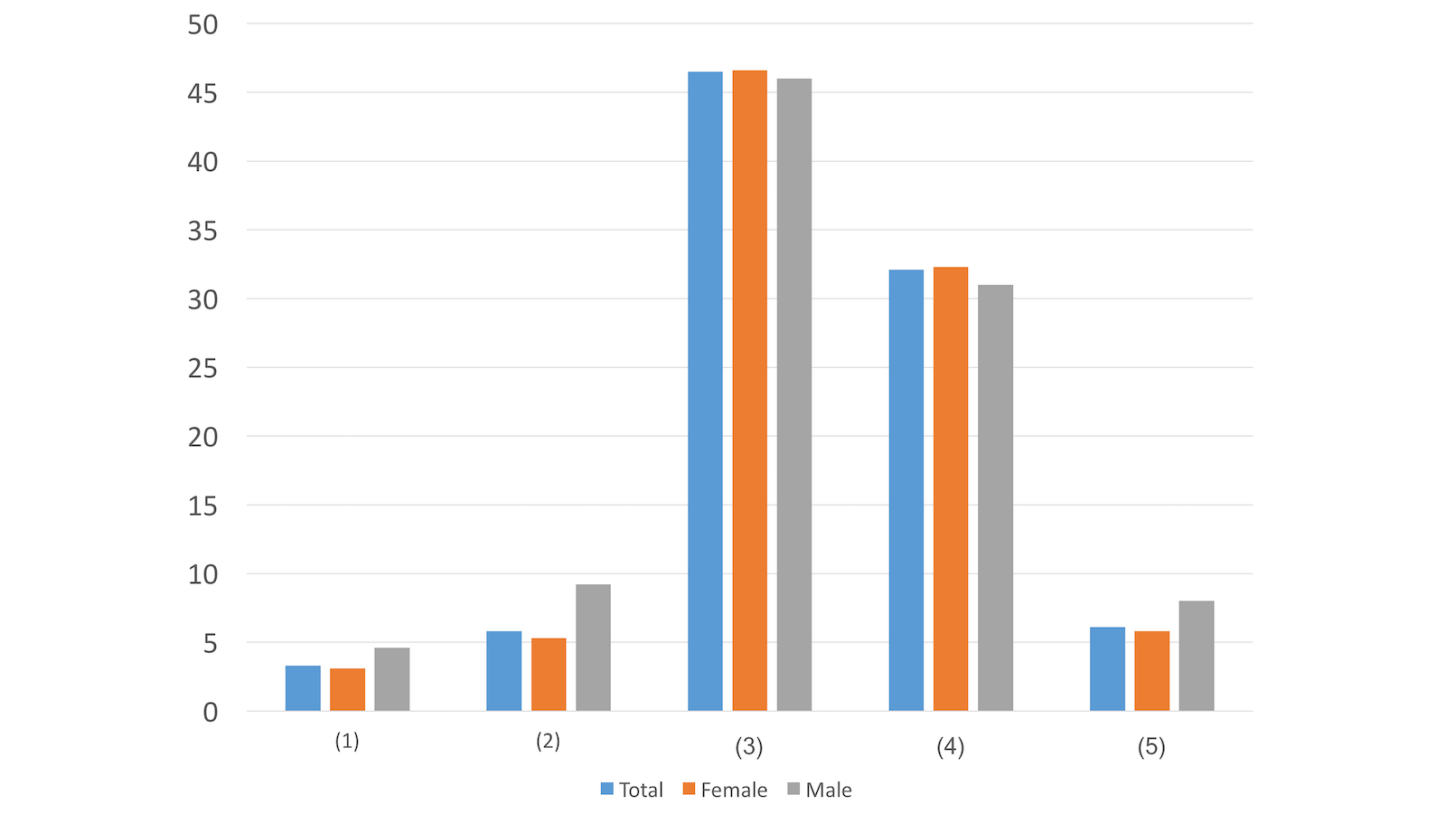
Results of study 2 question 9.

**Figure 10:**
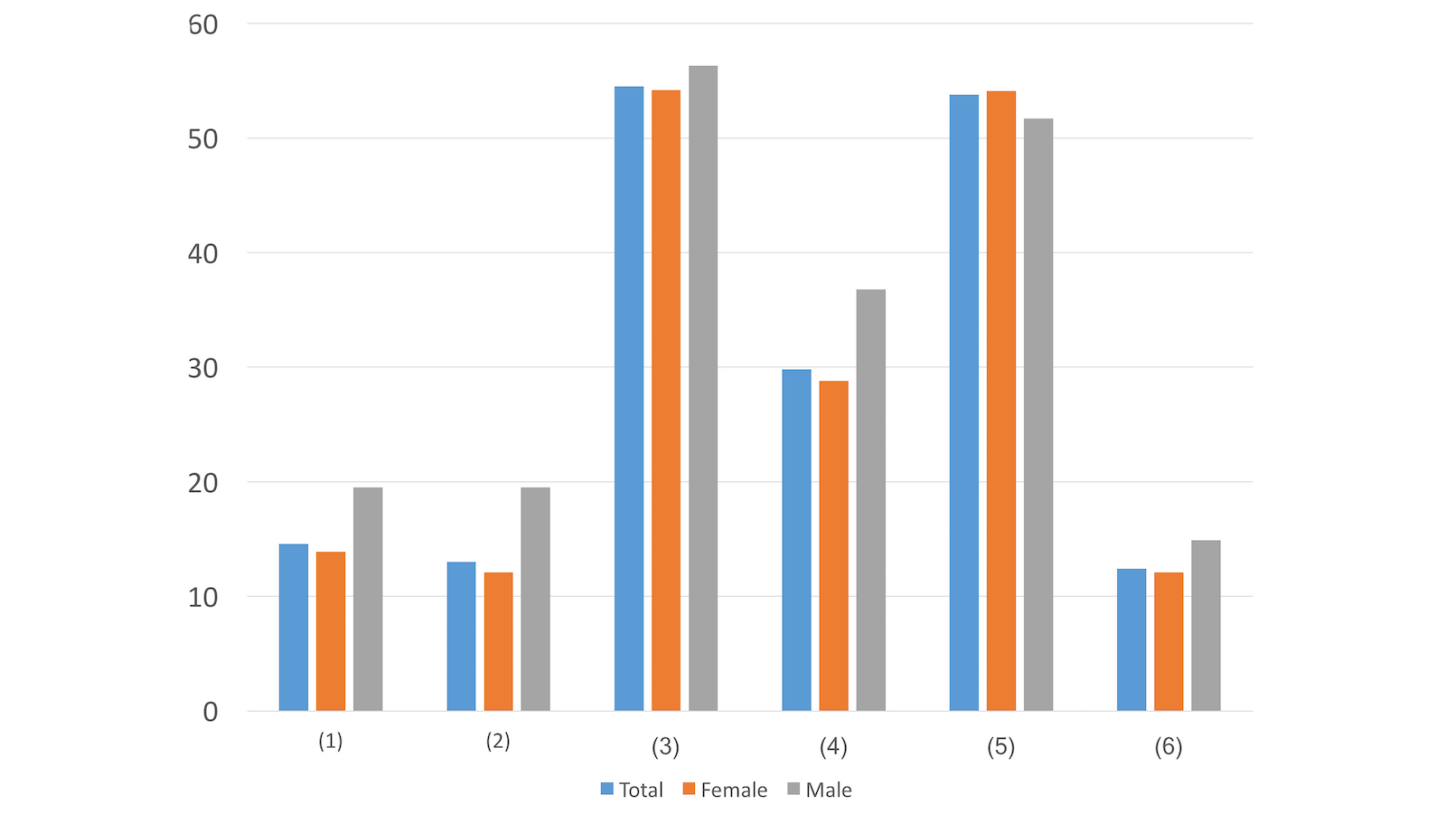
Results of study 2 question 10.

Of 4222 participant surveys, 725 (17.17%) were collected but some of the questions were left blank by certain participants. Before answering the 10-question survey, respondents also provided their background information with regard to suicide ideation and attitude toward suicide online intervention. A total of 88.0% (638/725) were female, and 12.0% (87/725) were male, with an average age of 21.22 (SD 3.69) years. More than two-thirds (493/725, 68.0%) of respondents had university or college degrees, and 93.3% (676/725) were unmarried. While 0.8% (6/725) of participants never had suicide ideation, 99.2% (719/725) had had ideation in the past and 51.2% (371/725) of participants had made suicide attempts; 78.6% (570/725) of the participants were not opposed to the idea of online suicide intervention. Figure 1 to 10 present the results of study 2.

#### Question 1

Question (Figure 1): Which account name do you prefer to click on and read the details when we send you direct messages for psychological intervention services?

1. Psychological Map
2. Psychological Assistance Community Group
3. Psychological Crisis Intervention Group
4. Life Protector
5. Walking Toward the Sunshine
6. Listening to Your Heart
7. Doesn’t matter
8. Other account names (please specify______)
9. Don’t want to read any direct messages

When choosing the most preferred account name, there were greater differences in options 6 and 9 compared to the others. While more males (14/87, 16%) chose the name Listening to Your Heart, only 10.5% (67/638) of females preferred this title. In addition, 6.9% (44/638) of the females tended to not read any direct messages but only 1% (1/87) of the males ignored them. These differences may suggest that males tend to be more interested in relatively indirect names than females do.

#### Question 2

Question (Figure 2): Which self-introduction method is more acceptable for you?

1. We read through your public Weibo posts and found that you were in a bad mood, even considering suicide...
2. Through searching keywords, our online intervention system finds that you are in a bad mood, even considering suicide...
3. Doesn’t matter
4. Other ways (please specify ________)

In terms of the self-introduction method, more males (22/87, 25%) prefer the online intervention system finding out their feelings than females (101/638, 15.8%) do. This may indicate that while females value others’ help, males tend to not seek help from humans and may feel more comfortable with computer assistance.

#### Question 3

Question (Figure 3): When you receive this direct message, do you care about the message’s account nature (eg, authority, nonprofit)?

1. Yes
2. No
3. Doesn’t matter

With regard to the message’s account, more males (39/87, 45%) than females (242/638, 37.9%) were interested in the nature of the account. This may suggest that males tend to be more wary and critical about the source of the information than females do.

#### Question 4

Question (Figure 4): At the beginning of this direct message, how do you prefer to be addressed?

1. Greetings
2. Dear Weibo User
3. Dear + your account name
4. Doesn’t matter

In this question, more males preferred general phrases like Greetings (28/87, 32%) and Dear Weibo User (4/87, 5%) compared to females. Meanwhile, females (131/638, 20.5%) preferred to be addressed with specific account names. This difference may suggest that males value privacy while females appreciate more help and attention.

#### Question 5

Question (Figure 5): Which status do you prefer the direct message account use when reaching out to you?

1. I’m a teacher from... (from a personal perspective)
2. We are... (from a team perspective)
3. Doesn’t matter

More males (25/87, 29%) prefer to be reached from a personal perspective, and more females (195/638, 30.6%) chose to be messaged by a team. This may indicate that while privacy is the relatively valuable factor for males, warmth and help from others are preferable for females.

#### Question 6

Question (Figure 6): In this direct message, which expression order do you prefer?

1. First state the account’s identity and nature, then express concern for you
2. First express concern for you, then state the account’s identity and nature
3. Doesn’t matter

Most of the population (269/725, 37.1%) preferred to learn the account’s identity and nature, then the concern for them. The main difference was in option 2. More males (27/87, 31%) than females (116/638, 18.2%) preferred the message to first express concern for them, then state the identity and nature of the account. This may suggest that the purpose of the message is relatively important for certain males, but the majority value the account’s identity and nature the most.

#### Question 7

Question (Figure 7): If the direct message contains a nonprofit psychological assistance hotline, how do you prefer the number to be presented?

1. If you need anything, you can call this nonprofit psychological assistance hotline: ####
2. This is a nonprofit psychological assistance hotline. Many professional counselors here are ready to listen to your story at any time: ####
3. Doesn’t matter

In this question, the main difference was in option 1. More males (15/87, 17%) than females (76/638, 11.9%) preferred the expression “If you need anything, you can call this nonprofit psychological assistance hotline” than “This is a nonprofit psychological assistance hotline. Many professional counselors here are ready to listen to your story at any time.” This result is in aligned with the result in Question 4 as males tend to prioritize their privacy before help and attention from others.

#### Question 8

Question (Figure 8): If the direct message contains a link, under what situation would you be the most likely to click on it and read the details?

1. As long as the account is reliable, I will click on it and read the details
2. The letter in this link might be helpful for you
3. Doesn’t matter
4. I never click on links

While more females (280/638, 43.9%) prioritized the importance of account reliability, more males (28/87, 32%) believed the content’s degree of helpfulness was positively associated with the possibilities of their reading the details of the content. This difference may indicate that women tend to be more willing to seek help as long as the account is reliable, while men may want to ensure the content is valid and helpful before they reach out for advice.

#### Question 9

Question (Figure 9): Which caption of the article in the link would draw your attention the most?

1. You only live once, and we try our best to help you get rid of any psychological crisis
2. Dig out the potential to let yourself have a better life
3. Maybe you are in a difficult situation, but there are always solutions for you
4. Doesn’t matter
5. Other options (please specify _______)

Most people (337/725, 46.5%) chose the caption “Maybe you are in a difficult situation, but there are always solutions for you.” While the participants may have felt neglected in the past due to pressure and discrimination around them, this option may make them feel valued.

#### Question 10

Question (Figure 10): What do you hope will be the content of the article? (You can choose more than one answer.)

1. Inspiring words
2. Inspiring stories
3. Specific suggestions for coping with psychological crisis
4. A nonprofit psychological assistance hotline for you to call
5. More content about self-understanding as well as feedback from psychological testing
6. Other (please specify____)

The majority of the respondents with suicide ideation requested the articles to include psychological assessment with feedback (390/725, 53.8%) and practical measures for crisis solving (395/725, 54.5%). The preference may suggest that the target population intends to seek specific advice and self-understanding skills as long as they are willing to click on the link.

## Discussion

### Principal Findings

In study 1, user preferences and attitudes toward direct messages as well as opinions on the prototype were reviewed. The nature of the intervention account received the most attention. The more reliable the account was, the more likely the provided link to extra information was accessed by the audience. In terms of the use of direct messages, they were employed mostly for purposes of comforting friends, greeting strangers, shopping online, etc. Direct messages were usually checked when notifications appeared, and a short text was mostly preferred. It was advised that the account name be changed to a more caring one in order for the audience to feel the support from the society. Another aspect to be changed was the content of the advice. It was suggested that the aim of direct message text to provide professional support and suicide intervention be highlighted. Communication with the patients was also emphasized to be conducted under privacy protection. Instead of a general text for the entire audience, a more customized design based on each individual’s situation tended to be preferred. Overall, phone numbers from the psychological intervention centers were preferred to be added to the content of the text.

The goal of study 2 was to survey a large population of microblog users with potential suicide ideation about acceptable engagement messaging for individuals with suicide thoughts. Most participants were not opposed to receive direct messages and showed interest in having psychological assessment with feedback from professionals as well as the practical measures for dealing with a crisis. Consistent with the findings of study 1, study 2 found that the more reliable the account was, the more likely the participants would click on the extra link. This consistency was of much importance because it emphasized the role of the account name’s reliability. Indeed, Langford and her colleagues [13] suggest that finding the target population’s “usual and trusted information sources and media usage” is crucial. The more comfortable audience members are with the sources, the more likely they will prefer to learn the content of the messages.

In addition, most people (46.5%) chose the statement “Maybe you are in a difficult situation, but there are always solutions for you.” While the participants may have felt neglected in the past due to pressure and discrimination around them, this option may make them feel valued. As shown in Langford’s study [13], the audience’s current perceptions are likely to shape their behavior. In this case, the participants may be shaped by their suicide ideation. If the audience feels the warmth from the society, they may be more likely to click on the link and feel less depressed after seeking help.

The majority of the participants (54.5%) chose specific suggestions for coping with psychological crisis in the article. Like in the previous statement, if they find that the caption of the article brought them warmth, they may prefer to learn more specific skills to cope with suicide ideation.

In terms of gender differences, males in the groups tended to be more critical about the account name than females, while females preferred to receive attention and help from others. This difference may indicate the various perceptions males and females had toward direct messages; males valued privacy while females appreciated the warmth from the society.

Despite the differences, the reliability of the account was positively associated with both groups’ tendency to open the link. It was also important for the text to contain psychological assessment with feedback for each individual. Paying attention to these details was crucial for designing an effective private message for the target population.

### Limitations

There were limitations to this project. The sample size was small, and gender distribution was biased. Future research should apply such messaging questions to a more male population as the male and female groups may have different responses as shown in study 2. Moreover, since the study was conducted on Sina Weibo, there could be a number of significant cross-cultural differences for generalizing the results to other populations (eg, Twitter in the United States). In addition, the interviews were not theory-based due to a lack of prior research for proactive online suicide interventions.

### Conclusion

We consulted with 2 groups of participants with self-reported suicide thoughts to help generate appropriate direct messages for help. Direct message is a vital function in Sina Weibo and may be used to reach any Weibo user with suicide ideation [7-11]. Receiving feedback from our participants was particularly important since we wanted to avoid messaging that would not draw attention and prevent people who had suicide ideation from seeking treatment. The results of the 2 studies showed that the account name’s reliability, brevity of the private chat, and details of the phone numbers of the psychological intervention centers as well as psychological assessment were associated with the attractiveness of the private chat. This paper provided 1 model for including target users in the development of direct messages for online suicide interventions.

Future research should include various explorations around messaging (eg, instead of sending out direct messages, a chatbot could be employed to interact with users) to determine how interventions might be delivered based on various message media. Larger samples in a future study could permit us to have a greater understanding of the differences between subgroups of the target population (depending on symptoms, preferences, etc) when delivering the interventions.

## Abbreviations

ASIQ-4: Adult Suicide Ideation Questionnaire
